# Not only laboratory to clinic: the translational work of William S. C. Copeman in rheumatology

**DOI:** 10.1007/s40656-020-00330-1

**Published:** 2020-08-06

**Authors:** Michael Worboys, Elizabeth Toon

**Affiliations:** Centre for the History of Science and Technology, University of Manchester, Manchester, M13 9PL UK

**Keywords:** Translation medicine, William Copeman, Rheumatology, Medical communication, Specialisation, Bench-to-bedside

## Abstract

Since the arrival of Translational Medicine (TM), as both a term and movement in the late 1990s, it has been associated almost exclusively with attempts to accelerate the “translation” of research-laboratory findings to improve efficacy and outcomes in clinical practice (Krueger et al. in Hist Philos Life Sci 41:57, 2019). This framing privileges one source of change in medicine, that from bench-to-bedside. In this article we dig into the history of translation research to identify and discuss three other types of translational work in medicine that can also reshape ideas, practices, institutions, behaviours, or all of these, to produce transformations in clinical effectiveness. These are: (1) making accessible state-of-the-art knowledge and best practice across the medical profession; (2) remodelling and creating institutions to better develop and make available specialist knowledge and practice; and (3) improving public and patient understandings of disease prevention, symptoms and treatments. We do so by examining the work of William S. C. Copeman, a dominant figure in British rheumatology from the 1930 through the late 1960s. Throughout his long career, Copeman blended approaches to “translation” in order to produce transformative change in clinical medicine, making his work an exemplar of our expanded notion of TM.

## Introduction

Since the arrival of Translational Medicine (TM) as both a term and movement in the late 1990s, it has been associated almost exclusively with attempts to accelerate the “translation” of research-laboratory discoveries into clinical applications (Krueger et al. 2019). Many discussions of TM assume a linear model, where “innovation starts with basic research, is followed by applied research and development, and ends with production and diffusion” (Godin 2006). One reason this model dominates is its applicability to pharmaceutical research and the goal of shortening, speeding up, or unblocking the “pipeline” of new chemical entities (NCEs) becoming approved drugs (Butler 2008). Observers and analysts have conventionally framed TM as a four stage process: T1—basic research to produce innovations with therapeutic potential, T2—development of clinical trials, T3—clinical implementation and uptake, T4—assessment of outcomes and effectiveness (Sung et al. 2003; Woolf 2008; Fort et al. 2017). But despite this initial framing, empirical studies have demonstrated the complexity of interactions between stages and that a fully linear process is rare. Historians’ studies of other areas of medical innovation have long shown that the same messiness applies (Pickstone 1992). Indeed, it is more normal for laboratory discoveries to “fail” to be translated into clinical practice, as demonstrated by the very low number of NCEs that become marketable drugs. Even when an NCE “succeeds”, the extent of its adoption and use will vary, as new drugs largely compete with other new therapies.

The point about “failed” innovations is a valuable counter to a common feature of studies of TM—namely, that they are teleological. Not only do they focus on “winners,” but the paths to success they describe can appear predetermined. However, in this study, we want to use one aspect of the teleological view constructively, to explore an expanded notion of TM, with TM referring not only to a *means* or *process*, but also to an *end* or *outcome*. In other words, a central goal of TM is transforming clinical practice and effectiveness. Highlighting this goal-oriented aspect raises the question: What of the other *means* of achieving the same *end*? In this article we discuss three other “translational” and “transformational” *means*: (1) Making available state-of-the-art, specialist knowledge and best practice to groups across medicine; (2) Creating or remodelling institutions to promote and develop knowledge and innovative practices; and (3) Developing ways to improve public and patient knowledge about disease prevention, symptoms and treatments. In the case we discuss below, all three routes involved translations of ideas, practices, institutions, behaviours, even all of these at once. Ideas were changed to work in new contexts and with new audiences, while practices were reworked to be effective in the hands of different groups. Institutions were remodelled to be more effective in achieving clinical outcomes, and professionals’ behaviours were modified to prevent disease or better manage treatments and aftercare.

The clinical area in which we develop our analysis is the treatment of patients with rheumatic diseases in England from the 1930s to 1970. Specifically, we explore how William Copeman, a dominant figure in the field in Britain from 1930 to 1970, led or orchestrated four translational and transformational activities—three in our expanded version of TM and one in the narrow bench-to-bedside mode. First, Copeman sought to transform general practice by disseminating best practice from specialists in a four-times updated handbook and instructive films. Second, he facilitated the establishment of rheumatology as an academic, research-led specialty in a four-times updated textbook. Third, he set out to make specialist rheumatological knowledge available in appropriate forms to the public in radio broadcasts, popular articles and books. Fourth, as we show, Copeman proved to be a pivotal figure in the development of innovations in the narrower sense of TM, helping to move a crucial, era-defining intervention—cortisone—from T1 to T4.

While we focus on Copeman’s efforts and impact, our goal is to use aspects of his story and his contributions to medicine to explore creatively what “translation” meant and now means—not to provide a comprehensive overview of Copeman’s career or of his role in the history of rheumatological research and practice. Our analysis therefore emphasises Copeman’s translational activities, by keeping the focus on his public work and roles as made visible to rheumatology and medicine. To do so, we draw especially on the published materials Copeman created and that reached diverse audiences: research articles, reports, textbooks, reviews, letters to journals, and popular media. Complementing this are archival sources (his personal and professional papers, along with those of societies where he played an instrumental role) that allow us to explore how and why Copeman set out to create these materials, and the effort involved in doing so. These materials allow us to show how and with what impact Copeman moved ideas and practices between contexts, and how the changes he made gained traction in those new settings.

## William Sydney Charles Copeman

Copeman was not directly responsible for any major breakthrough in the understanding or treatment of rheumatic diseases, but nonetheless, his peers would later agree that no individual had contributed more to the improved treatment of rheumatic diseases or the development of the speciality of rheumatology (Porritt and Hart 1992). In his notes towards a biography of Copeman, his friend and fellow pioneer rheumatologist, Arthur (later Lord) Porritt wrote “He had the rare gift, by virtue of his personality, of getting new and essential things done…. He was a great originator, a great producer” (Wellcome Archives n.d.). Copeman was best known for his public role in the Empire Rheumatism Council (ERC) and its successor organisation the Arthritis and Rheumatism Council (ARC), and for lobbying for the establishment of rheumatology as a specialism, but his impact proved to be wider. Throughout his career he was active in research, teaching, clinical care, publishing, speaking, broadcasting, lobbying and organising, all to further professional and public understandings of rheumatic diseases and to develop and extend treatments (Anon 1970a, b).

**Fig. 1:**
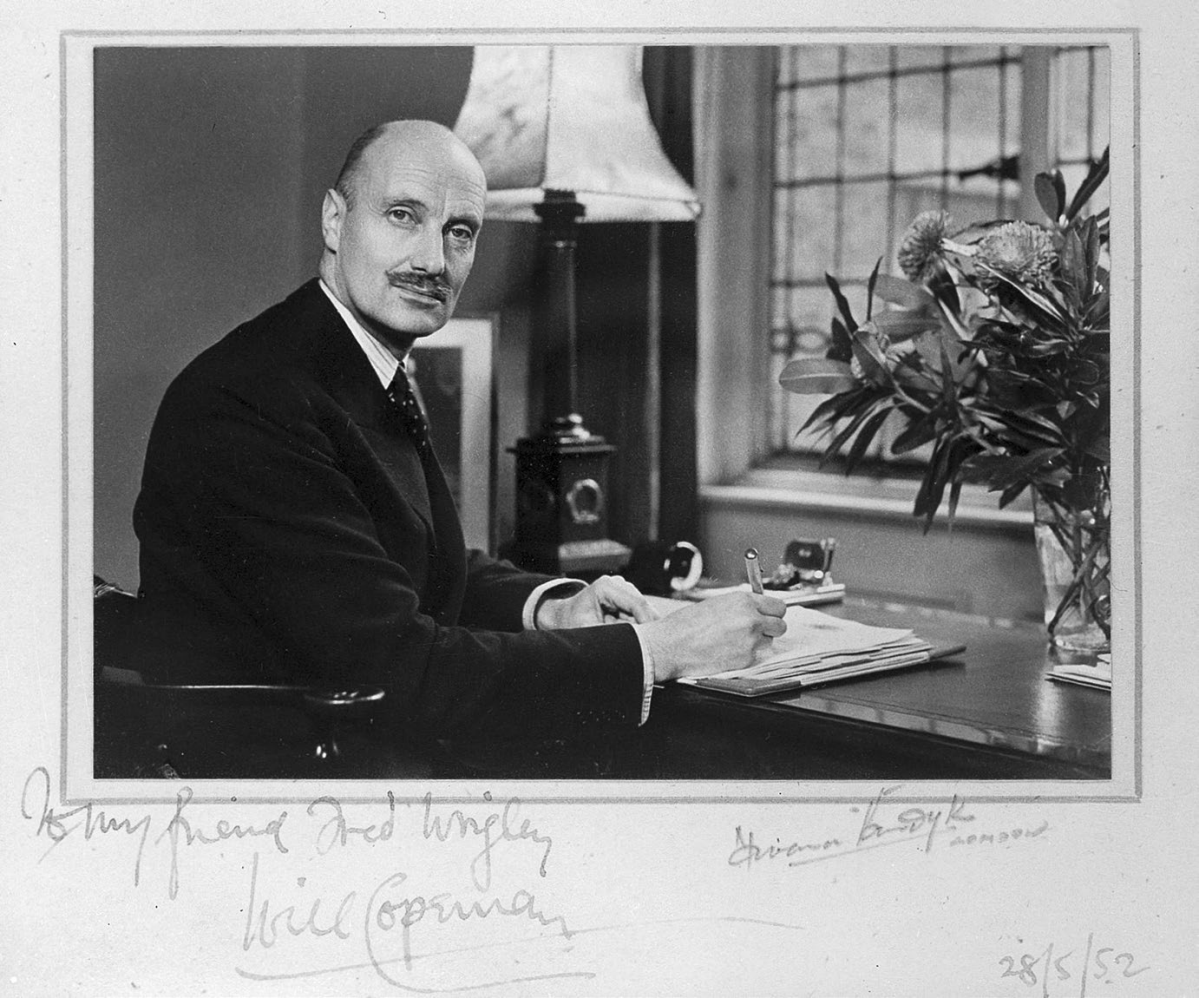
Portrait of William Copeman 1952. Seated at desk, with autograph inscription by sitter to F. Wrigley. Credit: Wellcome Collection. Attribution 4.0 International (CC BY 4.0)

Known to his friends and colleagues as Will, William Sydney Charles Copeman was born in July 1900 into an established medical family. His father was Sydney A. M. Copeman, the country’s leading authority on smallpox vaccination, who had also undertaken research on other infectious diseases for the Local Government Board (Copeman 2004). Will Copeman trained at St Thomas’s Hospital, taking the conjoint diploma in 1924 and graduating MB, BChir from Gonville and Caius College, Cambridge in 1925. His first appointments were in paediatrics, at the Sorbonne in Paris, then the Hospital for Sick Children, Great Ormond Street, London, and at the same time at the out-patient department at St Mary’s Hospital, London. His work on rheumatic diseases began with rheumatic fever in children and developed more widely when he was appointed to be Honorary Physician at the British Red Cross Clinic for Rheumatism in London, and Assistant Physician at the Hospital of St. John and St. Elizabeth. As was typical for elite London consultants in the 1920s and 1930s, he also held part-time or associate appointments at other voluntary and charitable institutions: The Home for Incurables, Putney, and the Star and Garter Home, Richmond, and then at the Middlesex Hospital when it took over Peto Place, and finally the West London Hospital. This spread of clinical experience and connections with patrons and governors was the basis for his private practice, based in prestigious Harley Street (Fig. 1).

His medical career, like that of so many of his age, took a different turn during the Second World War. He was given special duties regarding rheumatic diseases with the British Expeditionary Force in France in the war’s early period (Copeman 1940). However, after Dunkirk he served as a general physician, including acting as medical adviser to the Malta command, from where he published on meningococcal septicaemia and dyspepsia (Copeman 1942; Edwards and Copeman 1943). His interest in bacterial infections may have come from his father’s work, but more likely from the role that many doctors of the time felt that infections played in the aetiology of rheumatic diseases (Coburn 1931). In 1945, Copeman published a review of chronic rheumatic diseases during the war, noting that they had been neglected relative to other health problems. However, he maintained that “their ‘nuisance value’ was in the aggregate considerable, even producing a mild man-power problem in some regions, whilst the long average stay of hospitalised rheumatic patients aggravated bed shortages (Copeman 1946a).

After the war, and through the 1950s and 1960s, Copeman maintained appointments at the West London and Middlesex Hospitals, and at the Hospital of St. John and St. Elizabeth, along with his private practice. He also continued to publish extensively in journals and in textbooks that went through many editions; from the late 1940s, he added contributions to popular media, making the move into broadcasting. He even found time to write on medical history (Poynter 1971). Throughout this period, he also acted as the effective political leader of British rheumatology through his role in the Empire Rheumatism Campaign and membership of the National Health Service and Medical Research Council groups devoted to this specialty. As a de facto leader of British rheumatology, he also contributed to international organisations, as the first chair of the World Health Organization’s Expert Committee on Rheumatic Diseases (Anon 1970b; World Health Organization 1954).

## Specialist Practice to General Practitioners

The first of Copeman’s initiatives that could be called “translational” was his 1933 guide for GPs *The Treatment of Rheumatism in General Practice* (Copeman 1933). He did not lack confidence. At this point in his career, Copeman had at best only 3 or 4 years full-time rheumatological experience, yet he produced a book that aimed to translate and spread this newly-gained specialist knowledge to the nation’s general practitioners. His guide spoke to two implicit audiences: older practitioners whose knowledge was out of date; and new graduates who were likely to have seen very few rheumatic patients in their voluntary hospital-based training. Copeman was also clearly ambitious. He had already published in a number of journals on non-rheumatic subjects, such as measles, diabetes, scarlet fever, and varicose veins. It was only from 1930, just 5 years after graduating and coinciding his appointment to Peto Place, that he began to publish on rheumatic diseases. He announced his expertise in a review article in the *British Medical Journal* (*BMJ*) on “Some Principles in the Modern Treatment of Rheumatic Diseases,” which concluded that “it appears more and more obvious that the intelligent combination of remedies is the keystone of success, the danger lying in the adoption of any one method of treatment, to the exclusion of the rest” (Copeman 1930).

He appears to have first acted on his aim to transform arthritis sufferers’ treatment and care in a 1932 lecture delivered at the Royal Institute of Public Health, titled “The Control of Industrial Rheumatism” (Copeman 1932). There he set out a scheme for institutional innovations to improve the access of National Insurance (NI) patients, most of them working class, to specialist clinics and spas. At this time, as Cantor (1990) has shown, most cases of rheumatic disease were seen and treated by general practitioners, and not in hospitals. Patients fell into two groups. The first comprised middle and upper class sufferers, who before the NHS were mostly private patients who could afford to pay the general practitioner’s fees and then afford to be referred to specialists or make visits to spas. Meanwhile, working class sufferers (the second group) had a higher prevalence of rheumatic disease, because of the demands of industrial and manual labour, but had less access to spas and other treatment facilities with only the possibility of referral if an institution with appropriate specialists was nearby. In his discussion of rheumatism and the place of spas in interwar England, Cantor (1990) unpacks the conflict between the hospital and spa factions of professionals treating rheumatic diseases, showing that while they struggled over priorities and resources, most clinicians had a foot in both camps.

**Fig. 2:**
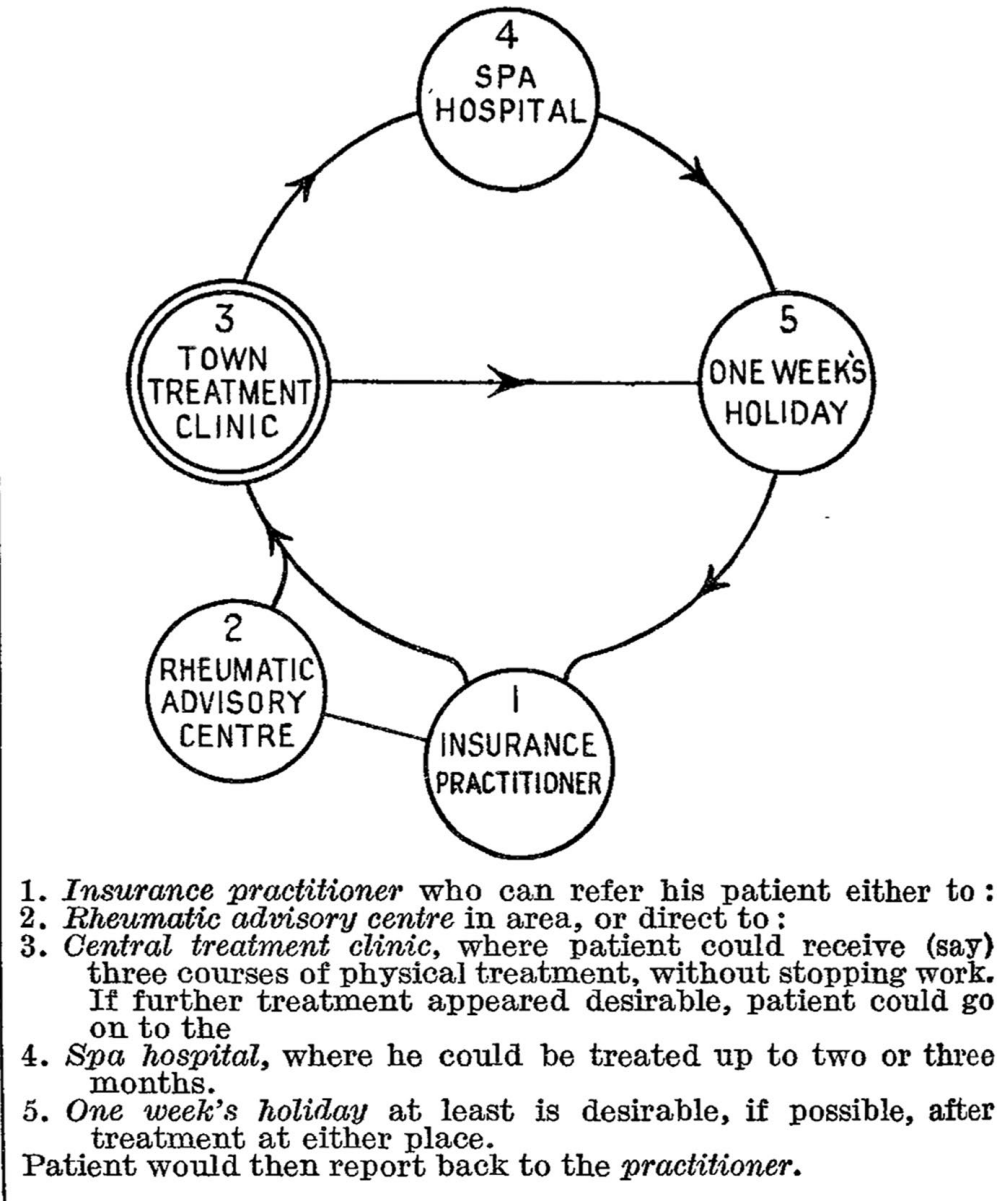
Diagram of scheme for a rheumatology service. Copeman (1932 p. 986)

Copeman’s plan to change the prospects of sufferers had three chief elements: prevention, detection and treatment. General practitioners treating sufferers with NI would play a primary role as illustrated in a diagram (Fig. 2).

Copeman argued that for these GPs treating national insurance patients, their role was diagnose, treat initially, and then route sufferers into a larger system of treatment that included new centres and clinics, while also providing the individual patient with “general advice, and encouragement”. The presumption behind this was the idea that GPs were slow or reluctant to make referrals, in part because they were (incorrectly) pessimistic about what could be done (Editorial 1932).

Copeman’s book *The Treatment of Rheumatism in General Practice* (1933) elaborated this plan further by promoting intra-professional communication. The first half of the book discussed the classification, presentation and prognosis of the many forms of rheumatic disease, while the second half went through the treatments available across the range of presentations. His aim here was not simply to disseminate specialist knowledge, but to make it understandable and usable for general practitioners and to do so in a succinct, accessible way that busy doctors might read. He would have had in mind that he was addressing some doctors who had qualified decades previously, even in the late nineteenth century, and that even those GPs eager to expand their knowledge had likely received little or no formal training regarding the rheumatic diseases. At first glance, Copeman might seem like a “know-all” elite physician talking down to “ignorant” general practitioners, a classic exemplar of the vision of science communication now widely known (and criticised) as the ‘deficit model’ (Turney 1998). However, his writing shows an awareness, no doubt gained from ample professional interactions with general practitioners, of their experience and understanding, and how to address GPs productively. For instance, in this volume he was forthright about the uncertainties that still dogged specialists hoping to better understand rheumatic diseases, a tactic that could well have won his readers’ confidence and cooperation.

The book’s early chapters focused on rheumatic fever and chorea, the childhood forms of rheumatism with which Copeman had first developed his interest in the area. However, he argued that these mostly acute conditions were distinct from the adult forms of rheumatic disease that were chronic and disabling. Chapters on muscular and neurological forms in adults came next, then the core of the book: the arthritic diseases of rheumatoid arthritis, osteoarthritis and ankylosing spondylitis. In the second half, Copeman focused on treatments, first with a chapter on “General Aims” and then with detailed discussions of medical, dietetic, and physical methods, baths, colonic therapy, endocrines, actino-therapy, and orthopaedics, plus advice on the choice of spas, doctor-patient relations, osteopathy and “nature” cures. He acknowledged that the treatment of rheumatism was characterised by conflicting opinions and that previously, in his own practice and writing, he had “at times been too dogmatic,” but he said his hope now was to present “a brief summary of all methods” and not to be exclusive. A final, novel feature of the book was a chapter on “Prognosis and End Results,” where Copeman hoped to convey the optimism underlying the new specialist units to general practitioners, thus converting them to the view that “rheumatism should no longer be considered as an ‘act of God,’ but may now be treated with a good prospect of success” (Copeman 1933, p. v). Reviewers for the leading general medical publications, the *Lancet* and the *British Medical Journal*, both noticed and applauded this optimism. The *Lancet*’s reviewer praised the book for being both approachable and stimulating: “it spreads an atmosphere of restrained enthusiasm which should encourage the practitioner to attack the rheumatic diseases with some hope of success.” Copeman was said to have shown critical and balanced judgment, giving clear descriptions of treatments which can be carried out in the home and those needing referral (Anon 1933a). The *BMJ*’s reviewer was similarly positive, stating that “Not the least valuable of Dr. Copeman’s recommendations is to be found in the phrase, “the doctor approaching an arthritic case must train himself to do so with real hope” (Anon 1933b).

No doubt spurred by these positive reviews, Copeman’s guide sold out in a few months, and a second edition followed 2 years later (Copeman 1935). By then, Copeman had competition, suggesting a broader disciplinary push to transform attitudes towards rheumatic diseases, with a number of ‘specialists’ putting their experience into print. George Kersley’s *The Rheumatic Diseases: A Concise Manual for the Practitioner* (1934) in William Heinemann’s *Practitioner* Series, also aimed to make up for the deficiencies of medical training, noting that graduates were more familiar with the rare diseases found in patients referred to hospitals with medical schools than the common illnesses of general practice. Matthew Ray, who also worked at the Red Cross Clinic, likewise published *Rheumatism in General Practice* (1934), with an endorsement from the well-known Lord (Tommy) Horder as “the best book on rheumatism in the English language.” Another Peto Place clinician, Francis Bach (1935), published on *The Rheumatic Diseases: Their Treatment and Recognition*, which was said to be more of a textbook, being “too full to appeal to general practitioners.” Last but not least in this rush into print came the more encyclopaedic *Chronic Rheumatism, Causation and Treatment* by Fox and van Breeman (1934). Clearly, Copeman had seen a market amongst GPs for both new knowledge about and new attitudes towards the management of rheumatic diseases.

The reviews of the second edition of Copeman’s book in 1935 again highlighted his ability to translate and communicate the complexities surrounding rheumatic diseases, which were very challenging conditions for both patient and practitioner. The anonymous reviewer in the *BMJ* noted that Copeman provided “a guide both as to what he can do for them within the scope of his own practice, and how he can maintain a reasonable sense of proportion in face of the astounding claims as to the efficacy of various nostrums which arrive by every post” (Anon. 1935). Copeman was quick to bring new ideas and treatments forward and in the two years between editions a number of new treatment modalities were added, including “gold salt therapy and short-wave diathermy.” He had drawn upon colleagues and specialists to update chapters, developing for himself a central place in professional networks that he utilised for his publications. In the third edition, Copeman (1939) introduced more new chapters on gout and focal sepsis,^1^ and even more new treatments employing physical therapy, new compounds, and new forms of radiotherapy. The rapid series of editions and the considerable changes made to each were themselves proof to readers that rheumatology was now a field of considerable therapeutic innovation, with expert leaders like Copeman willing and able to keep abreast of developments and communicate these to general practitioners. The fourth edition of *The Treatment of Rheumatism in General Practice* (1946a) also showed that knowledge change involved discarding theories as well as creating new ones: it was essentially a reprint of the third edition with the chapter on focal sepsis removed. (By the post-war years, the idea that infections such as tooth decay and sinusitis produced poisons with systemic effects had gone out of fashion.)

**Fig. 3:**
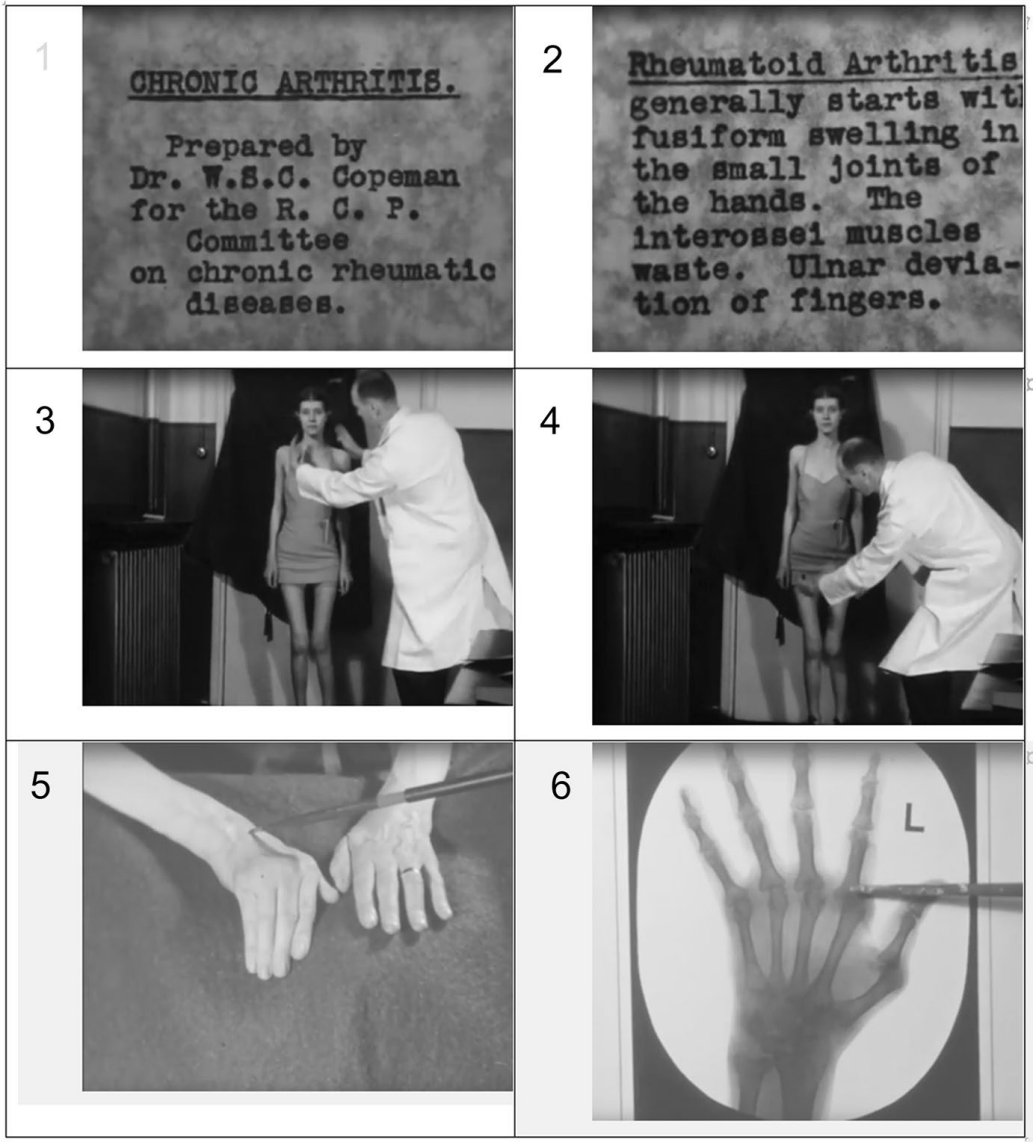
Stills from W. S. C. Copeman (1938a), *Chronic Arthritis, Prepared by Dr W. S. C. Copeman for the R. C. P. Committee on Chronic Rheumatic Diseases,* Wellcome Collection, Attribution-NonCommercial-NoDerivatives 4.0 International (CC BY-NC-ND 4.0) https://wellcomecollection.org/works/f8cujtz3. Accessed 13 July 2020

In the late 1930s Copeman adopted a new medium to communicate up-to-date ideas about rheumatism, by making a silent black and white film for the Chronic Rheumatism Committee of the Royal College of Physicians (RCP), mostly shot at the Peto Clinic (Copeman 1938a, b, c). Like other specialist-made medical films of the era, Copeman’s film focused almost entirely on diagnosis, shown in Fig. 3 images 3–6. Its intended audience was the general practitioner, as indicated by the use of medical terms in the captions, with the signs and symptoms of conditions illustrated by close-ups of affected joints, with features indicated by the clinician’s hand or pointer (Copeman himself was briefly in frame), backed up by X-rays and charts. The moving images of patients with different conditions imitated the style of instruction familiar in clinical teaching. The presentations were selective and stylised because of the brevity of the sections and the absence of sound. There were brief illustrations of possible aetiological factors, specifically jobs and activities that were associated with the diseases: a policeman directing traffic with arm signals, gardeners digging and sweeping, and even huntsmen riding with hounds. The first showing was at the opening of the Rheumatism Clinic at the West London Hospital on 2 February 1938, and the film was screened subsequently at meetings of medical societies, usually in association with lectures by Copeman (Report 1938; Copeman 1938b). The aim overall was again two-way translation: to make available expert knowledge to GPs and encourage GPs to refer patients to specialist units.

Copeman continued to reach out to general practitioners after the Second World War, first, as we have noted, with a new edition of his book and then in an article giving a refresher course for general practitioners on rheumatoid arthritis in the *BMJ* (Copeman 1952). The focus here was much more on treatment, now more widely available through the National Health Service, with recommendations for aspirin for the relief of pain, gold-salt therapy to control active disease, physical methods to improve mobility, and a cautious welcome for cortisone treatment (which we discuss in detail below). However, we must note here that soon after he also edited a book devoted entirely to introducing cortisone and ACTH to clinicians (Copeman 1953). The book’s coverage was wide, dealing with the value (or lack of it) of the new drugs in rheumatic conditions, plus their use in the treatment of diseases of the eye, endocrine system, respiratory organs, skin, blood and allergies. Lord Horder wrote in the Foreword that Copeman had again produced “a practical and concise account of the present position [and] most skilfully assessed the value of these substances and their association with other forms of treatment” (Copeman 1953, p. i).

## Rheumatology as an Academic Specialism

An explicit expectation of Copeman’s translational strategy was that better informed general practitioners would transfer more of their patients to specialist units like his own. However, he was only too aware that there were few “rheumatologists” (as the specialists were coming to call themselves) and specialist units, and that recent improvements in the treatment of rheumatic diseases had not been anywhere near as great as in many other areas of medicine (Weisz 2006). Thus, the development of rheumatology as an academic specialty was a corollary of his pitch to GPs, but it was important to him that rheumatology be known as a research-oriented, academic subject. In the 1930s, two organisations served as the primary vehicles for promoting of rheumatology as an academic specialty and transforming the specialty’s scale and standing. These were the ERC and the Heberden Society, and Copeman played a leading role in both. The ERC had been founded in 1936 with the seemingly omnipresent Lord Horder as chairman (Horder was at the same time also active in British cancer organisations and with several general medical bodies). William Willcox served as vice-chairman, and Copeman acted as medical secretary. In practice, this meant he effectively ran the Council day-to-day, organising meetings, establishing its public profile, and running its public appeals for funds (Witts 2004). The ERC’s formal aims sounded quite narrow and academic: “to organize research throughout the British Empire into the causes and means of treatment of rheumatic disease.” However, its leaders interpreted these aims broadly, to include making the best means of treatment available to all sufferers (Report 1936). The previous year the RCP had appointed a Committee on Chronic Rheumatic Diseases, which then produced reports for 1934, 1935 and 1936; these reports brought together a number of new research papers, many written by Copeman, and called further for the expansion of treatment facilities (Buckley 1935, 1936, 1937). Those behind this initiative developed links with the ERC, principally through Copeman, and with the American Committee for the Control of Rheumatism, through Philip Hench at the Mayo Clinic who was its secretary.

Some of the elite London physicians involved with the ERC and the RCP Committee supported efforts to establish “rheumatology units” in teaching and voluntary hospitals, though others fought to keep rheumatic diseases in the ambit of general physicians. Copeman, not surprisingly, supported the specialist-led version of practice, and successfully created two such units, first in 1937 at St. John and St. Stephens and in the following year, at the West London Hospital. These began as out-patient departments but soon acquired beds for a limited number of inpatients. The treatments they offered were spa-like, but there was less emphasis on water and more on physical methods such as massage, manipulation, splinting, and electrical stimulation. They were sites also for experiments with medical treatments, as with gold therapy on which Copeman published in 1936 and 1937 (Copeman 1936; Copeman and Tegner 1937). However, these new sites were few in number. In 1938 in England, a few of the hospitals associated with medical schools had rheumatism clinics, and in rest of the country’s hospitals, which numbered over 1000, there were just fifty specialist rheumatism departments (Empire Rheumatism Council 1939).

The origins of the Heberden Society were in the Committee for the Study and Investigation of Rheumatism that had been formed at the Peto Place Clinic in March 1936, on which Copeman was a prime mover (Copeman 1942). The society’s name was chosen to honour William Heberden (1710–1801), the physician who set out one of the earliest descriptions of rheumatism and gout. The Society initially held its quarterly meetings at Peto Place, where attendees read and discussed papers on clinical aspects of rheumatic diseases (Moll 1987). These meetings were interrupted by the war, but overall continued until 1983 when the Society was absorbed into the British Society for Rheumatology (Savage 1988).

The character of the Society’s early meetings was later described by Jonas Kellgren, who would himself pioneer academic rheumatology in Manchester (Dixon 2010). Kellgren pointed to difficulties in communication, deriving from differences in orientation and approach, between some members:

My earliest recollection of the Heberden Society was a meeting in 1938. This was held in a small room in Peto Place, Dr. Heald in the chair was clearly a distinguished elderly physician and Will Copeman stood out from the rest as the keen able young physician in immaculate pin stripes and black coat. The remainder of the audience of some dozen people seemed to be elderly spa doctors who asked some rather odd questions and it seemed at this time most unlikely this group would develop into the fine scientific and clinical society that we have today (Moll 1987 p. 76).

We can see in this description the broader generational, geographical, institutional, and therapeutic divides amongst those treating rheumatic diseases. Stereotypically these can be characterised as young-London-hospital- and chemo-biological-research orientated (Kellgren clearly perceived himself to be one of those) *versus* old-provincial-spa-practice and physical-treatment orientated. Copeman, though, would play a mediating role, despite being London- and hospital-based, with a Harley Street practice and association with elite physicians at the RCP. He was the leading figure in the Chemical Sub-Committee of the RCP’s Rheumatism Campaign (RCP 1938), chaired by Lionel Whitby (Gardner and Tansey 2004), who had pioneered chemotherapy for infections with sulphonamides. Popular with his peers, Copeman had a record of referring his patients to other clinics that were trialling new treatments, such as gold and vaccine therapy, and was involved in distributing the ERC’s research funds (Chemical Sub-Committee 1938).

The division between urban, science-minded researchers and provincial spa-oriented practitioners was often overdrawn by those involved for political reasons and while Copeman sometimes wrote of two distinct approaches to rheumatic diseases (Copeman 1933), in practice he worked across them. For instance, he recommended both medical and physical methods in all editions of his book for general practitioners, arguing for the value of both: “One of the most important therapeutic advances in this century is the discovery that the human body can be influenced as much from the outside by what are known as Physical Methods as from the inside by Medical Methods” (Copeman 1946a, b). Despite later being known for his work around cortisone and other high-profile drugs, Copeman always advocated the use of occupational therapy in the treatment of arthritis and furthermore was active in the British Association for Physical Medicine. After the Second World War, physical medicine in Britain was reoriented to have a greater emphasis on rehabilitation, first with injured combatants, and then with industrial workers (Tegner 1972). This reorientation was mirrored by the name of the society’s journal: first in 1942, the *British Journal of Physical Medicine* added “and Industrial Hygiene” to its title, and then in 1947 this was replaced this with “including its Application to Industry.” Francis Bach, who also worked at St Stephen’s Hospital, edited a review of the field in *Recent Advances in Physical Medicine*, drawing on the expertise of 38 contributors (Bach 1950). His review featured sections on public health, and on the health of defined populations (school children, the Army, and industrial workers), as well as on rehabilitation and resettlement. In his review of Bach’s work for the *BMJ*, Copeman (1951) strongly supported Bach’s call for a new type of “physical-medical specialist,” in what he saw as an “infant specialty.”

Copeman himself, though, was not to be a member of this “infant specialty”. Rather he increasingly committed himself to the development of rheumatology as a disease-defined, academic-medical specialty, one that was based primarily in medical schools and hospitals. He hoped such a specialism would overcome existing divisions and raise the status of the field. A common sign of aspirations like these is the creation of a journal, and Copeman also engaged in this. In the mid-1930s, the Annual Reports of the RCP’s Committee had served as a nascent journal, and when these ceased, the ERC took over and carried on the mission formally. In 1939 the first volume of *The Rheumatic Diseases* was published, though its title would soon become *Annals of the Rheumatic Diseases*. This new journal was edited by Charles W. Buckley, in collaboration with Copeman and Alfred G. Timbrell Fisher and published quarterly (Anon 1955a, b; W. A. L. 1968). Buckley was in one sense a traditional spa doctor, in that he worked at the Devonshire Hospital for Rheumatic Diseases in Buxton, but unusually he was also Fellow of the Royal College of Physicians and active in the ERC. Timbrell Fisher, meanwhile, also straddled the supposed gulf between spa doctors and urban specialists: an orthopaedic surgeon at St Stephen’s Hospital, in the1930s he had published on the viral origins of rheumatic disease, but he would be best known for his work on the manipulation of joints (Fisher 1991). In the journal’s early years, its articles focused chiefly on the aetiology of rheumatic conditions (especially possible bacterial and viral causes), their pathology and morphology, and on serological markers of disease. There were no articles on treatment, suggesting a research rather than clinical focus. The journal continued to be published through the Second World War, and its editorial team would soon be supplemented by Mervyn H. Gordon and two American associate editors: Philip Hench and Loring Swaim.

The Second World War provided Copeman and others like him with the opportunity to publicly, professionally and politically promote rheumatology as a clinical specialism of national importance. It also brought challenges, as when in 1939, Copeman was put on general medical duties rather than expected to continue with his specialist work. Nevertheless, in July 1940 he gave a talk, “Notes on Treatment of the Rheumatic Diseases in the B.E.F,” that continued to push his vision for rheumatology’s organisation. There, he called for centralised, specialised units to provide the best treatment for troops on the frontline in order to avoid conditions becoming chronic and loss of personnel. He also argued that such units offered economies of scale, and “what is perhaps of greater importance, it would stimulate interest in this type of case, which does not exist widely to-day, in spite of the high incidence of these diseases” (Copeman 1940, pp. 384–385).

An important part of the ERC campaign was its annual publication of *A Plan for National Action*. This at last bore some fruit when, thinking about post-war health service planning, the Ministry of Health set up a sub-committee on rheumatic diseases. Hopes were thus high, after the passage of the National Health Service Act in 1946, that when the new service began rheumatology would enjoy new support and status. After all, in the years leading up to and after the start of the NHS, there were many reports by professional bodies and government committees on the need to develop rheumatology services. However, there ended up being no central direction for these efforts and it was left to regional and local bodies to act. Some did establish departments or centres, but most did not, with the innovators being in northern cities (Leeds, Manchester, Sheffield) and in familiar London hospitals (West London, Hammersmith, Royal Free), but in most places hopes that specialist posts or specialist departments would be created were dashed (Horder 1953). Those calling for specialist posts and units faced opposition from those working in physical medicine and their technique-based practice, and from general physicians as the development of drug treatments had normalised the management of rheumatic diseases to their style of practice.

In 1945, *Annals* became the official publication of the Heberden Society, with the editorial committee remaining the same and with Copeman joining a new Editorial Committee in 1947. In marking its formal association with the ERC, Buckley, who was then the President of the Society, stressed that now “its primary object [was] the study of the clinical aspects of rheumatic disease,” but saw no conflict with the research orientation of the ERC. Indeed, Buckley questioned “whether any group of diseases has suffered more from methods of treatment devised without any consideration of the underlying pathology responsible for the symptoms presented” (Buckley 1945). None the less, the Society was social as well as expert, being quite traditional. It had special regalia, many types of honours, high class dinners, and a historical library. It was the ideal arena for networking and Copeman was a central figure, making its library a great resource and professional hub.

Copeman further cemented his place as the leader of academic rheumatology in 1948 when he brought together what he termed “a galaxy of talent” to contribute chapters to a *Textbook of Rheumatic Diseases* (Copeman 1948). The edited volume covered all forms and all aspects, from aetiology and pathology, to prevention, treatment and aftercare. At the centre of the book were eleven chapters on individual rheumatic diseases, which dealt again with all aspects of each condition. There were just four on physical medicine: i.e. radiotherapy, physiotherapy, hydrotherapy and spa treatment. The contributors were the leading clinicians in the field and mostly (seventeen out of twenty-four) from London hospitals. Only three of the remaining seven contributors were from provincial spas, including Buxton’s Buckley and Bath’s George Kersley. Copeman wrote the introductory chapters on nomenclature and classification and the history of the disease, and two other clinical chapters on chorea and fibrositis. Reviewing the text for the *BMJ*, Kenneth Stone highlighted its presumed audience, noting that it was “intended to be a textbook for those young medical men who have decided to embark on a specialist career in the rheumatic diseases” (Stone 1948). However, his assessment, no doubt coloured by the recent publication of his own book *Diseases of the Joints and Rheumatism* (Stone 1947), offered only faint praise: while Copeman’s textbook was “a very good book of its kind” and “superbly produced, profusely and well-illustrated,” Stone wrote, with twenty-four authors it was “not a textbook but a symposium, with the inevitable repetitions and sense of discontinuity” (Stone 1948). Other reviewers disagreed: when the second edition was published in 1955, the *Journal of Bone and Joint Surgery*’s reviewer praised the volume as “an outstanding achievement” (Capener 1955).

The major addition to this second edition was endocrinology, where a new chapter by Oswald Savage reflected “the major advances which have resulted from the discovery of the action of Cortisone, Hydrocortisone and ACTH.” As Copeman explained in the preface, these major advances had developments also led to “the complete rewriting of several chapters,” indicating that the new approaches were being incorporated and translated across many areas of practice (Copeman 1955, p. v.). These efforts paid off: one reviewer noted that the volume had “deservedly secured its place as a standard textbook,” another regarded it as “the” reference work (Anon 1955a, b; Lawrence 1956). The new steroids as well as other drugs and compounds, such as gold, phenylbutazone, and aspirin, were considered across several chapters. This shift rebalanced the textbook’s content to decisively emphasise medical over physical approaches, and foretold a laboratory-led, pharmaceutical future for therapy.

The shift to medical approaches continued in the third edition of Copeman’s textbook in 1964. A review by Jeffrey (1965) noted that, “Compared with its predecessor, the third edition of Dr. Copeman’s textbook has grown by 80 pages and half a pound in weight,” despite the use of smaller type. Jeffrey welcomed the timing, as the innovations in treatment of the late 1940s and 1950s now had “fallen into reasonable perspective.” The chapter on radiotherapy was withdrawn, there being “no well-defined field for such therapy with the rheumatic diseases.” Copeman’s comprehensive coverage meant that the chapter on hydrotherapy remained, though largely unaltered, while there were new chapters added on auto-immunity and genetics. This breadth made the Copeman textbook, in its many editions, stand out as a contribution to the field and not only in Britain. In 1970, Gabor Inke, a New York anatomy professor, sent a questionnaire sent by to medical schools to determine “the most frequently recommended medical textbooks” (Inke 1971). Respondents mentioned the third edition of Copeman’s textbook, one of only three volumes (of thirty-four listed) to cover rheumatology.

The fourth edition of the textbook (Copeman 1969) was produced just 4 years later, prompted by the need to add “further advances … in the fields of biochemistry, auto-immunity, cytology and genetics,” plus changes in surgery and new epidemiological information. There was a changing of the guard, with many stalwart contributors absent, notably H. A. Burt, Mervyn J. Gibson, B. Schlesinger, William Tegner and Sir Reginald Watson-Jones, who had all contributed to the three previous editions. The replacements were the new elite: D. L. Gardener, head of the Kennedy Institute, Allan St. J. Dixon, Clifford F. Hawkins, Stephen Mattingly, Emmanuel Miller and James T. Scott. Still contributing nearly 20 years on were H. Osmond Clarke, Henry (now Lord) Cohen, D. V. Davies, Campbell Golding, Jonas Kellgren, George Kersley, Oswald Savage, and the editor. While much was new, Copeman retained his chapter on non-articular arthritis rheumatism, even though the last cited reference was to a paper published in 1954. Indeed, the condition was becoming an increasingly contested diagnosis; on the one hand it was being redefined as fibromyalgia and on the other its status as a rheumatological condition was disputed altogether. One view was that it was a “medically unexplained somatic syndrome,” that belonged to mental health rather than rheumatology. Indeed, its status continues to be debated in the twenty-first century as the “fibromyalgia wars” (Wolfe 2009). Nevertheless, the durability of Copeman’s textbook in academic rheumatology was evident when two further editions, with his name in the title, were published after his death (Scott 1978, 1986). By that point, Copeman’s drive to remake rheumatology as an academic, research-led specialism, one committed to producing new specialist knowledge that could be translated to general practitioners and employed in everyday practice, had been achieved.

## Rheumatism and the public

In 1947, Copeman had been one of the founders of another organisation lobbying for the support of rheumatic disease: the British Rheumatism Association (BRA), meant as a complement to the ERC. BRA allowed public membership and set out to perpetuate the pre-war tradition of voluntarism in convalescence, by founding what were called “Horder Homes” (to teach sufferers how to cope best with their condition) and by recruiting “Horder Helpers” to work between home and hospital. But the Association focused mostly on attracting public attention to pain relief, physical treatment, and assistive devices and services for the rheumatic patient, as evidenced by at an early meeting (September 1949) where:

Some 15 firms had stands displaying preparations for the relief of rheumatic disorders, electro-medical apparatus, invalid furniture and transport, and devices for occupational therapy. There were also displays illustrating spa treatment, home and after care visiting, and industrial rehabilitation (Report 1949).

Copeman, however, struck a somewhat different note at this meeting, by speaking enthusiastically about a laboratory-sourced treatment, which he referred to as an “epoch-making announcement.” This was, of course, Compound E, soon to be renamed cortisone (Report 1949), and its announcement would (in retrospect) lead the takeover of pharmaceutical approaches in the treatment of rheumatic diseases. But Copeman did not only promote cortisone; he also made the most of the opportunities the announcement of this “epoch-making” drug opened for promoting a new rheumatology. He did so in two ways: first, by building on the news splash around cortisone to develop new opportunities for public engagement, considered in this section, and second, by leading the clinical trials facilitating introduction of the drug in Britain, which we consider afterwards.

On 3 May 1950, billed anonymously as “a doctor,” Copeman (1950a) began a series of eight 15-min weekly talks on “Rheumatism” for the BBC’s Home Service. (This anonymity was expected by professional ethics at the time, as doctors were supposed to eschew anything that smacked of advertising or naked self-promotion, though they could speak on behalf of their organisations. This would begin to change a decade later.) The “Rheumatism” series was a didactic “public information” medical broadcast typical of those given in the late 1940s and early 1950s (Karpf 1988; Loughlin 2005; Nathoo 2009, 33–56; Jones 2013). The talks existed in the first place due to Horder’s long-time lobbying, as he had broadcast radio appeals for support of rheumatic disease (and other diseases) from the late 1920s onward (Horder 1928). Copeman’s stated aims, though, were somewhat different. He focused not on raising funds but on allaying the fears of sufferers and giving hopeful grounds for relief; on informing a larger public of the scale of the rheumatism problem and the need for early investigation of any symptoms: and on promoting the ERC as the organisation responsible for shaping public discourse about rheumatism. The first talk, “What is rheumatism?” promised the listener he or she would learn “what can be done for you, and what you can do yourself if you are a sufferer.” The seven following broadcasts addressed the main areas set out by the RCP Committee in the 1930s (rheumatic fever and heart disease; fibrositis, sciatica and neuritis, rheumatoid arthritis, osteoarthritis, arthritis of the spine and gout, and rheumatism research) and then Copeman finally asked “What of the future?” The talks were gathered in a pamphlet which was published by the ERC in August 1950, and then reprinted a further five times that year.

A virtue of Copeman’s broadcasts was his empathy with the suffering of affected listeners, which was likely to have been common as, at the end of the first broadcast, he highlighted that three million working weeks were lost every year, with these enduring and frustrating conditions. In the third talk, Copeman discussed lumbago or backache, the most common and popular terms for “rheumatism,” which he explained was fibrositis—a condition of muscles rather than joints. The complaint was associated with particular occupations that involved prolonged lifting or bending, or “may come on for no apparent reason—in you or me as we jump blithely out of bed one morning.” Relief might be spontaneous and if not, he recommended aspirin or bathing in hot water. Recognising how few homes had bathrooms, Copeman set out other domestic “physical” remedies that did not require access to a bathtub: “place the patient on his face and put a piece of brown paper over his back. With this to protect his skin a really hot domestic iron can be run over his back to ‘iron out the pain’ as they say.” In persistent cases, doctors might recommend massage, electrical treatment, injection of local anaesthetics, support plasters, or a belladonna plaster. To lighten the narrative, Copeman turned to history, noting the impact of the condition on British history: Wellington was only in command at Waterloo because he had been sent back from India where he had been “a martyr to lumbago.” The next talk was on sciatica (slipped disc) and neuritis, followed by three talks on arthritic conditions. In the final talk on “Rheumatism Research—What of the future?”, he discussed cortisone, bemoaning how it had been boomed by the press when it was still in trial and supplies were very limited. Copeman endeavoured to give listeners a better appreciation of medical research as he understood it with a military analogy. He said that the experimental work on cortisone had “established a deep ‘beachhead’,” but time was needed for this to link up with “the main body” of medical knowledge (Copeman 1950, p. 46). His entrance into broadcasting was a success, and opened further doors: in subsequent years, Copeman became a regular on the Home Service, giving talks on rheumatic diseases in programmes such as “Science Survey,” “European Science,” and “Woman’s Hour” (Copeman 1950–1954, Box 6).

In 1954, with Richard Mason, Copeman returned to print with a popular book on rheumatism for Duckworth’s popular *Modern Health* Series, edited by Thomas Horder. The series aimed

to give precise, authoritative information; to take the patient into the doctor’s confidence …; to better explain simply and intelligibly; to offer sufferers whatever hope the most up-to-date medical knowledge can honestly offer; in short to tell the truth about disease as it has never been told to the general public in print before. (Horder 1954–1957)

This description included several “translational” actions: “to better explain simply and intelligibly,” “to offer sufferers … the most up-to-date medical knowledge,” and “to tell the truth.” The other books that launched the series were similarly framed and were on subjects as varied as heart disease, varicose veins, and epilepsy; others followed on tuberculosis, skin diseases, cancer and allied diseases, childbirth, diabetes, and foot troubles. Readers were reminded the books were “designed to give general background” and were “not courses of self-treatment”.

The opening epigram in Copeman’s and Mason’s book was from the writing of the orthopaedic surgeon G. R. Girdlestone and reiterated the importance of morale as well as medicine in treatment: “Don’t try and live as though you were quite well, but never lose hope. It isn’t what happens to you that matters. It’s how you take it!” (Copeman and Mason 1954, p. 8) However, the overall message of the text itself was much more positive about the possibilities of a new and research-led medicine: there was much that medicine could do, especially if sufferers sought early treatment. Indeed, Copeman and Mason stated, it was vital “to dispel the most damaging factor—the feeling that nothing can be done.” Again, the aim was to convert sufferers into patients, describing common symptoms and interpreting to be treatable by modern medicine. The discussion of each rheumatic condition was similar to that in his 1950 talks, though there were more historical references, reflecting Copeman’s growing interest in the subject. The final chapters were the most practical, on “What the National Health Service Provides” and “’Gadgets’ Which Help.” Unsurprisingly given his position in the ERC, Copeman was clear that while the NHS now offered sufferers more than was available before 1948, it was not enough and there was much more both government and charities could do.

When he succeeded Horder as the head of the ERC in 1955 and remained at its helm when it became the Arthritis and Rheumatism Council (ARC), Copeman became the public face of British rheumatology. He fronted most the Council’s appeals for funds in print, on the radio and eventually on television, asking for donations to continue its work in transforming the lives of sufferers. Together with the BRA (which later became the BRAA or British Rheumatism and Arthritis Association), the ARC launched “Arthritis Month” in June each year to raise the public profile of its work. Copeman’s strategy for gaining attention and support was to translate patient suffering into an economic cost, which brought headlines such as: “£80 Million Lost from Rheumatism,” “‘Startling Figure’ on Arthritis,” and “Rheumatism’s Toll of Work Time” (Report 1961, 1962a, b). He also lost no opportunity to lobby for rheumatology more widely, especially for it to be given NHS resources. In 1969, he queried the spending priority given to heart transplants, contrasting the high profile (and high costs) of these interventions with the relative neglect of rheumatic diseases, which were much more common, caused greater suffering, and would give greater returns for many more sufferers (Roper 1969). Four years earlier, he had even seized on a discussion about the government’s decision to find the money to keep a Cezanne painting in the country, noting that by contrast the government gave little attention to research on rheumatic diseases (Report 1965). In other words, throughout this pivotal period Copeman either found or created new opportunities to promote his vision of how rheumatological knowledge could be produced, transferred, and applied. Whether he directly addressed the public or engaged in political lobbying, he spent his time translating his clinical knowledge and experience to practical ends, endeavouring to “sell” the value of rheumatology at all levels, from the individual up to the economy.

## Laboratory to clinic

So far we have focused on the importance of translational activities other than from laboratory to clinic. But now we turn to briefly consider Copeman’s work in that area, which was very significant indeed. As mentioned above, Copeman’s move into radio broadcasting had been linked to a clinical research project, namely working with cortisone. He led British trials with the drug, which as a number of scholars have shown, was regarded as the most important turning point in the treatment of rheumatic diseases in the twentieth century (Rodnan and Benedek 1970; Marks 1992; Cantor 1992, Hetenyi and Karsh 1997; Dixon 2000; Benedek 2011, Haller 2012). But Copeman was in the prime position for this role because of the activities discussed above. It was also an opportune moment. In his history of British rheumatology, Dixon (2000, p 15) suggests that 1948 was an *annus mirabilis* for the specialism, citing the general optimism due to the creation of the NHS and the wider availability and impact of antibiotics, along with specific developments such as the discovery of the rheumatoid factor (a diagnostic antibody) and the introduction of the anti-inflammatory drug Irgapyrine, which contained a future therapy for arthritis, phenylbutazone (Worboys and Toon 2018). However, in hindsight the most important events were the early reports from the United States of compound E, developed by Philip Hench at the Mayo Clinic.

Copeman had had previous contact with the Mayo group, especially from 1942 onwards when Hench, who published frequently in the journal, became an associate editor of *Annals*. Most significantly, the October 1948 issue of *Annals* had included the oration Hench gave to the Heberden Society that year, in which he reviewed the work that led to the investigation of adrenal hormones and compound E. Later, the June 1949 issue of the *Annals* had a scoop: it was the first mainstream rheumatology journal to reproduce the paper announcing compound E, a paper that had previously only appeared in *Proceedings of the Mayo Clinic*, a publication that was not widely available. An accompanying editorial, most probably written by Copeman, presciently predicted that, “Apart from its effect in treatment, [cortisone] will stimulate research in many directions which may produce results of the utmost importance” (Editorial 1949).

Copeman and Oswald Savage, his colleague at the Middlesex Hospital, visited the Mayo Clinic in 1949 and 1950 to learn about compound E. On the second occasion, Savage was given “a small supply” by Hench, which was used in the first English trial, in a study funded by the Medical Research Council and the Dan Mason Research Foundation of the West London Hospital (Copeman et al. 1950). The American findings were confirmed: the drug gave remarkable relief of symptoms, returning good mobility to sufferers previously immobilised. However, it only relieved symptoms; it was not a cure. Symptoms returned when the drug was withdrawn, which in some cases had been prompted prematurely by adverse reactions (Worboys and Toon 2019).

Copeman’s group at the Middlesex published a number of articles on the new drugs based on cases they had treated (Copeman et al. 1952; Copeman and Savage 1953). More significantly, based on this experience, Copeman was made chair of the Medical Research Council’s steroid committee, which controlled the distribution of the limited supply of the available drugs and supported clinical trials, adopting the then-novel double blind clinical trial protocols advised by Austin Bradford Hill (Bradford Hill 1952; Marks 1997; Bothwell and Podolsky 2016). The main trial, supported by the Medical Research Council (MRC) and the Nuffield Foundation compared cortisone and aspirin, the latter “another drug usually regarded as efficacious in relieving symptoms and in improving the patient’s functional capacity” (Joint Committee 1954). The conclusions, published in May 1954, were surprising to say the least. The trial found that “For practical purposes … there appears to have been surprisingly little to choose between cortisone and aspirin in the management of these 61 patients in the early stages of rheumatoid arthritis” (Joint Committee 1954, p. 1227). This bombshell finding was reported widely in the national press under headlines such “Aspirin Maybe Best” and “Aspirin Equals Cortisone” (Anon 1954a, b). A second report published in September the following year, confirmed the findings on equivalence of cortisone and aspirin (Joint Committee 1955). These were important findings as cortisone, while relieving symptoms, produced a number of serious side effects, whereas the benefits and risks of aspirin were well known. The controlled and limited introduction of cortisone, due initially to shortages in supply and costs, turned out not to have been as disadvantageous to British patients as previously thought, as the main alternative appeared just as good but with fewer risks.

The response of the pharmaceutical industry to the problems with the supply and toxicity of cortisone was to search for new versions, with fewer risks and greater efficacy in mitigating symptoms (Worboys and Toon 2019). These efforts were successful and led to a new class of drugs, the corticosteroids, the most favoured of which in the late 1950s were prednisone and prednisolone. Copeman and his colleagues at the West London Hospital conducted trials on these new drugs, not so much with RCTs but clinic-based, case reports on groups of patients, following on from trials in the United States. Meanwhile, Copeman continued to lecture and publish across medicine, as he had for the public sphere, on corticosteroids, speaking as the country’s leading clinical researcher and research organiser on the subject. His full and detailed reports to these various audiences were themselves acts of translation: he translated clinical research results into terms that helped his peers understand the benefits and risks of this new class of drugs, particularly by comparative framings with other treatments and with cases from his own clinical practice.

Nearly a decade on from the debut of compound E, Copeman reported that with rheumatoid arthritis, steroid therapy was only valuable in the minority of cases that did not respond to “classical and conventional methods, including physiotherapy” (Copeman 1958). Similar studies in centres across the country came to the same conclusion (Duthie et al. 1955). Nonetheless, Copeman explained that with certain patients “steroid therapy can make the difference between crippledom and painless independence,” providing doctors closely supervised patients to monitor side-effects. He was less positive about prednisone and prednisolone than many of his colleagues, but was confident that further research would produce “a substance whose action is confined entirely to the requisite therapeutic properties, without unwanted side-effects” (Copeman 1958, p. 176).

Copeman’s many roles enabled him to continue to foster research, but leadership was taken up by a new generation. In October 1963, he gave up the Chairmanship of the ERC, being promoted to the position of President (Report 1963). The new Chairman, Viscount Knollys, oversaw immediate changes. The ERC became the Arthritis and Rheumatism Council (ARC) and there was a new push to fund laboratory research, with a major investment in the Kennedy Institute of Rheumatology, then at the West London and Charing Cross Hospitals (Report 1964). In 1961, Mrs Mathilde Kennedy, the younger sister of Sir Simon Marks and part-heir to Marks and Spencer, gave £500,000 [£12 M in 2019] to fund a research centre in rheumatology (Report 1962a, b). Copeman led attempts to find a host institution in London, which proved difficult as most regarded such a centre as a burden rather than a boon. The Charing Cross Hospital agreed to take it on, only to renege on the promise. Eventually, the Hammersmith Hospital agreed, stipulating that a revenue stream of £200,000 over 5 years be found. A request was made to the ARC and turned down. Copeman then intervened, using his new post a chair of the Council’s Research Committee to secure the necessary support. The Kennedy Institute opened in October 1966, with a two-page spread in the *Times*, with, unsurprisingly, a lead article by Copeman, and others by Savage, Duthie, Lord Knollys and Dugald Gardiner, the Institute’s first director. The Institute occupied a newly built, six-storey building with clinical research facilities, including an out-patient clinic, and laboratories for morbid anatomy, biochemistry, immunology, bacteriology, cellular biology and experimental pathology (Dixon 2000; Anon. 1964). Copeman remained chair of the Kennedy’s management committee until his death in 1970.

## Conclusion

This article has used the career and work of William Copeman to contend that there are important “translational” activities in medicine other than those of laboratory origin, and thus that the hegemony of the “bench to bedside” shorthand for TM can limit our understanding of the sources of change in clinical medicine. Copeman’s efforts highlight the importance of the translation, transfer and transformation of knowledge and practice in three neglected areas: from specialists to general practitioners; from researchers to students and practitioners through the development of academic specialisms; and from doctors to patients and the public. Perhaps most importantly, for Copeman and for others like him, all these forms of translations came as a package, where each supported and extended the others. We discussed Copeman’s work in what would traditionally be considered TM, bench-to-bedside work on cortisone and corticosteroids in clinical application and trials (T2), at the end of this article, because his story reminds us that translation of clinical knowledge does not necessarily begin with a single drug embodying a concept, but with the activities of those who produce knowledge through and around it, and who in most cases have been doing so for some time before the drug appears.

It is impossible to give a quantitative estimate of the impact of Copeman’s activities in the translations and transformations that we have discussed. This is because all involved multiple, interacting factors that changed over time, in part as a result of Copeman’s goals of transforming all aspects of practice in rheumatology. However, his enduring leadership role in many organisations would suggest that he was highly influential. In their appreciation of Copeman’s importance in contemporary medicine, Porritt and Hart write that “He was seldom entirely sure he was in the right, would ask others their opinion and … usually act upon it.”^2^ In terms of rheumatology as an academic specialism, both his handbook for general practitioners and his textbook sold well, each going through four editions. For nearly 40 years he was the most important gatekeeper in British rheumatology, though the term does not do his work justice. He did much more than control the flows of knowledge, he tailored the presentation of ideas and practices to suit the needs and interests of different groups. Nor was he alone in this kind of work: readers familiar with the mid-century history of biomedicine and medical specialism will likely know of other professionals who occupied similar roles in other areas (for instance, see Cantor 2002; Toon 2007 on cancer in Britain). Copeman exemplified an era in clinical research where in efforts to produce new knowledge about disease and new structures for investigating and treating disease overlapped and interacted with efforts to produce new public understandings and experiences of disease.

The 1950s were Copeman’s most influential decade, when he was active with all four types of translation. Due to his standing already, from two decades of leadership, he was well placed to take a leading role in introduction of cortisone. In his writings on the subject, he was always clear that bench to bedside was never a singular or simple process, rather there had to be “continual investigation,” which we would term translation and retranslation, to determine the most effective and safest treatments, in a landscape where patient profiles, available drugs and trials protocols were constantly changing. From the 1950s, Copeman’s activities became increasingly focused on the ERC and seeking the establishment and expansion of rheumatology in the NHS, with the goal of a network of clinicians and specialist departments covering the country. In the years leading up to, and after the start of the NHS, there were many reports, by professional bodies and government committees on the need to develop rheumatology services, almost all of which were about encouraging the transfer of patients from GPs to specialists and better informing GPs and the public of the possibilities of the new specialism. At the end of the 1950s, there had been little progress (Dobson 1958; Copeman 1958). A further 10 years on, Copeman was still complaining about the absence of special units for a class of diseases that caused, arguably, the greatest suffering and economic losses for individuals and the country (Report 1967a, b). Rheumatology as an academic specialism, aided by dedicated ERC and ARC funding and pharmaceutical innovations, had flourished, but rheumatology as a clinical specialism remained marginal. The reasons for this lie beyond this study, but Copeman’s translational activities give clues. He was always emphasising the personal, social and economic costs of rheumatic conditions, the implication being that chronic diseases, which disproportionately affected the elderly and women, had not moved to the centre of medical policy, and were yet to attract public investment they deserved. In hindsight, we can say that the end he was seeking was the transformation of health services and medical work to best meet the challenges of a new world of disease, one where chronic diseases had become much more important.

While we have advocated a wider conception of “translation” and “translational” activities in medicine, our analysis can still be seen in terms of the conventional four-stage TM process. We are, however, arguing for much more focus on clinical implementation and uptake—T3, in other words for the transformation of clinical practice and effectiveness. Studies of industrial innovation no longer frame “implementation and uptake” teleologically as passive and one-way, but as active adoption, involving adaptions and change, with reciprocal impacts on practice at all levels and on social relations and institutions. Thus, Copeman’s first translational endeavours with general practitioners had the goal of changing the knowledge and culture of general practice. They aimed to make doctors better informed, open to change, more confident in referrals, and more optimistic with patients about treatment. The promotion of academic rheumatology was about establishing research-focused cadres of specialists, keeping medical schools and hospital practice up to date and encouraging innovation. Finally, Copeman believed an informed public would be more willing to seek early treatment and comply with advice, benefiting themselves and making doctors’ task easier. This would have increased demand for specialist services and, of course, should have encouraged donations to charities. In short, studies of Translational Medicine and “translation” in medicine should not confine themselves to documenting and promoting processes of technical change. Instead, we think they would profit from a wider view of the many, multiple kinds of work that have been necessary to accomplish transformation.

## Data Availability

This work was supported by the Wellcome Trust and will be Open Access. Images in Figs. 1 and 3 are from the Wellcome Collection. Figure 2 is an image made by the authors and their copyright, to be CC-BY.

## Abbreviations

ARD: Annals of the Rheumatic Diseases
BMJ: British Medical Journal
ERC: Empire Rheumatism Council
IRC: International Congress Rheumatology
JAMA: Journal of the American Medical Association
NA: National Archives (Kew, London)
WA: Wellcome Archives

## Footnotes

1 Focal sepsis refers to the common early twentieth-century view that rheumatic diseases had their origins in a localised infection and that from this toxins spread to produce systemic disease (Mollison 1920; Glover 1930; Hughes 1994).

2 Porritt and Hart, “W. S. C. Copeman,” (1992, 285).
